# A protocol for a systematic review of the diagnostic accuracy of blood markers, synovial fluid, and tissue testing in periprosthetic joint infections (PJI)

**DOI:** 10.1186/s13643-015-0124-1

**Published:** 2015-11-02

**Authors:** Paul E. Beaule, Beverley Shea, Hesham Abedlbary, Nadera Ahmadzai, Becky Skidmore, Ranjeeta Mallick, Brian Hutton, Alexandra C. Bunting, Julian Moran, Roxanne Ward, David Moher

**Affiliations:** Division of Orthopaedic Surgery, University of Ottawa, 501 Smyth Rd., Ottawa, ON K1H 8L6 Canada; Knowledge Synthesis Group, Ottawa Hospital Research Institute, Center for Practice-Changing Research, 501 Smyth Rd., Ottawa, ON K1H 8L6 Canada; Ottawa Hospital Research Institute, 501 Smyth Rd., Ottawa, ON K1H 8L6 Canada; University of Ottawa Faculty of Medicine, 451 Smyth Rd., Ottawa, ON K1H 8L1 Canada; Bruyère Research Institute, 85 Primrose Ave., Ottawa, ON K1R 6M1 Canada; School of Epidemiology, Public Health and Preventive Medicine, Faculty of Medicine, University of Ottawa, Ottawa, ON Canada

**Keywords:** Diagnostic accuracy, Systematic review, Periprosthetic joint infection, Joint replacement, Knee, Hip, Shoulder

## Abstract

**Background:**

Total joint replacement (TJR) procedures have been one of the most rewarding interventions for treating patients suffering from joint disease. However, developing a periprosthetic joint infection (PJI) is a serious complication that is associated with the highest burden of cost and reduction in patients’ quality of life compared to other complications following TJRs. One of the main challenges facing clinicians who are treating PJIs is accurately diagnosing infection in a timely fashion. Multiple orthopedic associations have published clinical guidelines for diagnosing PJI which are based solely on consensus approaches, expert opinions, and narrative reviews. We believe that a higher quality of scientific rigor is necessary to establish a diagnostic guideline that represents current evidence more accurately and that identifies important knowledge gaps in PJI diagnosis. Therefore, we will conduct a systematic review on diagnostic performance of blood markers, synovial fluids, and tissue tests for diagnosing PJI.

**Methods/design:**

Electronic search strategies will be developed and tested by an experienced medical information specialist in consultation with the review team, and gray literature will be searched using the checklist from CADTH’s Grey Matters Light. Two reviewers will independently screen the literature for inclusion using the prespecified eligibility criteria. Non-English language and animal-only studies will be excluded. Quality assessment and data extractions by reviewers will be verified, and disagreements will be resolved through consensus or third party adjudication. We will assess the quality of individual studies using the QUADAS-2 tool and use GRADE to summarize the strength of body of evidence. Analyses of evidence will be conducted in accordance with the *Cochrane Handbook for Diagnostic Test Accuracy Reviews*.

**Discussion:**

We will conduct a systemic review of tests (blood markers, synovial fluids, and tissue testing) for diagnosing PJI in patients’ knee, hip, and shoulder joint replacements. This will be the first scientifically rigorous and comprehensive systematic review in the field and may feed into an evidence-based clinical practice guideline. We will compare the findings of this review with the consensus-based guides and discuss the differences, similarities, and knowledge gaps.

**Systematic review registration:**

PROSPERO CRD42015023768

**Electronic supplementary material:**

The online version of this article (doi:10.1186/s13643-015-0124-1) contains supplementary material, which is available to authorized users.

## Background

Total joint replacement (TJR) procedures have been one of the most rewarding interventions for treating patients suffering from joint disease. However, developing a periprosthetic joint infection (PJI) is a serious complication that is associated with the highest burden of cost and reduction in patients’ quality of life when compared to other complications following TJRs [1]. One of the main challenges facing clinicians who are treating PJIs is accurately diagnosing infection in a timely fashion. Multiple orthopedic associations have published clinical guidelines for diagnosing PJI which are based solely on consensus approaches, expert opinions, and narrative reviews [2–5]. Furthermore, they fail to cover all types of prosthetic joints.

The average rate of PJI within 2 years after primary TJR is estimated at 2.0 %, but can reach as high as 14 % in revision TJR surgeries [6]. The current process of diagnosing and treating PJIs incurs a substantial clinical and economic burden for surgeons, hospitals, and most importantly, patients [7]. Based on an economic analysis by Kurtz et al. [8], the annual cost to US hospitals of treating PJIs has increased from $320 million to $566 million over the span of 8 years and is projected to exceed $1.62 billion by 2020. Establishing an accurate and a timely diagnosis of PJI is a key step toward implementing an effective treatment. An earlier diagnosis with an associated surgical intervention does lead to improve survivorship of the implants (i.e., no recurrence of infection and preservation of the original implants thus significantly reducing the morbidity and cost). Additionally, late diagnosis of PJI results in biofilm formation by the infecting organism. This biofilm coats the whole implant surface which necessitates the extraction of the whole implant in order to eradicate the infection. This procedure is very morbid and costly. The development of biofilm also makes the infecting organism resistant to antibiotic therapy. Biofilm formation starts within the first 2 weeks of infection symptoms; therefore, establishing and early diagnosis of PJI is critical to improve patient’s outcome and response to therapy [9, 10]. We can improve the quality of care delivered to patients suffering from PJI by translating the knowledge synthesized from this systematic review into evidence-based recommendations that can guide clinicians and surgeons in accurately and quickly diagnosing PJI. These recommendations also constitute the cornerstone for further research to fill the knowledge gaps identified in PJI diagnosis.

Patients who develop PJIs are predisposed to multiple surgeries that can lead to the loss of their life or limb and result in longer than average lengths of stay in the hospital. A major clinical challenge in successfully treating PJI is the current lack of “gold standard” diagnostic tests or protocols for accurately diagnosing infection in a timely fashion. To date, there are two main tests proposed as gold standard by recent guidelines for assisting clinicians in their diagnosis of PJI: sampling synovial fluid or tissue to assess the prevalence of neutrophils and culturing the offending organism, and testing serum levels of C-reactive protein (CRP) and erythrocyte sedimentation rate (ESR). Having said that, the sensitivity of synovial fluid culture is challenged by the so-called culture-negative infections. Currently, there are no clear clinical pathways to diagnose PJI and to describe when and how these diagnostic tests should be used. The current status is the presence of few consensus guidelines that recommend the use of blood markers, synovial fluid, and tissue testing to guide the clinician in diagnosing PJI. However, these current guidelines lack the evidence of indicating how these tests should be triaged and added on or used to replace an existing test [2–5]. We believe that this systematic review can help address this gap by identifying the true evidence supporting the diagnostic performance of each candidate test chosen. These results will help guide clinicians to develop a clear clinical pathway for the use of these tests based on evidence. The eventual purpose of these tests is to rule out infection and/or confirm infection and not to identify those at risk of developing a PJI. As such, these markers as stand-alone tests would be optimal.

Numerous studies have shown that these diagnostic tests are limited in their accuracy [11]. Although synovial or tissue cultures yield 95–100 % specificity, they have poor sensitivity of only 56–75 % [12]. The CRP and ESR are non-specific markers of inflammation and therefore are not reliable during the first 3 weeks of the postoperative period or in the event of other inflammatory conditions such as rheumatoid arthritis.

The literature reports over 20 additional diagnostic tests developed to improve the sensitivity of detecting early signs of PJI [13–20]. These investigations are diverse but fall into four main categories of testing, namely, serum, synovial fluid, synovial tissue, and nuclear imaging.

Studies that describe the benefits of these tests are heterogeneous in their designs and recommendations which limits their clinical applicability. Additionally, numerous guidelines published for diagnosing PJI are based solely on consensus approaches, expert opinions, and narrative reviews [2–5]. In addition, some of these new biomarker tests are more expensive and not readily available in most laboratories. We believe that a higher quality of scientific rigor is necessary to establish a diagnostic guideline that represents current evidence more accurately and that identifies important knowledge gaps in diagnosing PJI. Therefore, we propose a research question: “what is the current evidence for the accurate diagnosis of PJIs using published protocols that support the use of blood markers, synovial fluid and tissue testing as diagnostic tools?” Our objective is to conduct a systematic review that can assess the current evidence for the diagnostic accuracy of diagnostic tests available for PJI. Nuclear imaging, however, will be excluded from the protocol because the test, although highly sensitive, is shown to be non-specific with consequently moderate to poor reliability [21, 22].

The goal of this review is to generate new quantitative evidence for clinicians and guideline developers to establish evidence-based guidelines for diagnosing PJI. Ultimately, this will improve the management of patients with PJI, as effective treatment of PJI requires accurate and quick diagnosis.

## Methods/design

The methodological approach to evidence searching and synthesis, as described in this protocol, will conform to the Cochrane Collaboration’s diagnostic test accuracy methods [23]. Our approach consists of performing a literature search, screening the studies identified, and selecting the studies that meet the eligibility criteria. We will extract the data from the selected studies, assess their methodological quality, and perform a GRADE assessment on the body of evidence. Other activities will include statistical analyses, evidence synthesis, and report compilation that will be carried out in chronological order as outlined in the steps below. We will adhere to standards of the Preferred Reporting Items for Systematic Reviews and Meta-Analyses (PRISMA) in reporting the findings of this review [24]. The content of this protocol follows the Preferred Reporting Items for Systematic Review and Meta-Analysis Protocols (PRISMA-P) recommendations [25]. (Please see Additional file 1 for PRISMA-P detailed checklist.) This review is registered with the International Prospective Register of Systematic Reviews (PROSPERO) [26]. The registration number is CRD42015023768.

### Eligibility criteria

Table 1 presents the detailed eligibility criteria for this review. English language studies and studies with all sex- and gender-eligible (post-arthroplasty) populations reporting data from disease (PJI) of the hip, knee, and shoulder that investigated one of the candidate laboratory tests (see below) will be included. Therefore, animal-only studies and studies that do not report data on diagnostic performance of candidate index tests against the clinical reference standard will be excluded or removed.

**Table 1:** The review eligibility criteria

Study characteristics	Inclusion criteria	Exclusion criteria
Population	Patients who have undergone knee, hip, and shoulder joint replacements (no time frame as infections may be chronic)	Patients who have undergone joint replacements for other joints; and studies that use animal testing
Index tests (intervention tests)	Blood markers, synovial tests, and tissue culture tests:	Imaging tests
	Blood markers	
	*• Serum C-reactive protein (CRP)*	
	*• Serum erythrocyte sedimentation rate (ESR)*	
	• Blood culture	
	• Serum white blood cell (WBC) count	
	• Serum interleukin-6 (IL-6) levels	
	• Serum procalcitonin levels	
	• Serum interferon-alpha levels	
	• Serum toll-like receptor 2 (TLR2)	
	• Human beta-defensin-3 levels	
	• Neutrophil CD64	
	• Soluble intercellular adhesion molecule-1 (sICAM-1)	
	• Tumor necrosis factor-alpha (TNF-alpha)	
	• Neutrophil elastase 2 (ELA-2)	
	• Bactericidal/permeability-increasing protein	
	• Neutrophil gelatinase-associated lipocalin	
	• Lactoferrin (LF)	
	Synovial fluid tests:	
	• *Synovial fluid white blood cell (WBC) count; Synovial fluid polymorphonuclear neutrophil percentage (PMN%) analysis*	
	• *Synovial C-reactive protein (CRP) levels*	
	• Synovial neutrophil-derived circulating free DNA/neutrophil extracellular traps (cf-DNA/NETS)	
	• Intra-articular purulence	
	• Application of synovial fluid to leukocyte esterase test strip	
	• *α*-*defensin*	
	Tissue tests:	
	• *Periprosthetic tissue culture/swab culture/joint aspiration culture*	
	• *Histological analysis: PMN per high power field*	
	• Gram stain	
	• Routine acid-fast bacillus (AFB) testing and fungal testing	
	• *Polymerase chain reaction (PCR) and molecular techniques*	
	Note on how the above tests were selected: We first selected the three main diagnostic categories that are considered to be important in identifying PJI (blood markers, synovial fluid tests, and tissue tests). Under each major category, we chose the tests that are routinely used and are considered part of good clinical practice such as ESR, CRP, and tissue cultures. We have also included new diagnostic tests, such as alpha-defensin and leukocyte esterase strips, that have been recently published and described as novel tests that can overcome some of the limitation of the routine tests.	
Reference test (comparator test)	Joint fluid (with cell count, gram stain, and culture) or tissue (with histopathology and culture)	
	Note: given the lack of gold standard for diagnosing PJI, we will also consider any gold standard used in the primary studies and consult our clinical experts to accurately classify them.	
Outcomes	• Sensitivity and specificity of individual tests	Studies that do not investigate diagnostic performance of the index tests
	• Negative predictive value (NPV) and positive predictive value (PPV)	
	• Positive likelihood ratio (LR+) and negative likelihood ratio (LR−)	
Study designs	We will include randomized controlled trials, cross-sectional, systematic reviews, controlled cohort, and case-control studies (recognizing that the latter may overestimate test performance) [37–39].	Case reports, commentaries, expert opinion, and narrative reviews

Index tests in italic font are the tests prioritized as the primary tests that we will use to assess their diagnostic accuracy performance. The rest of the index tests are considered as secondary to our analyses, and depending on the amount of literature and work load, it may or may not fall under the scope of this review to assess their diagnostic accuracy performance. Table 1 presents the eligibility criteria (inclusion and exclusion), particularly population, index tests, reference test, and outcomes of interest for this systematic review

*α* alpha, *AFB* acid-fast bacillus, *Cf-DNA* circulating free DNA, *CD64* cluster of differentiation 64, *CRP* C-reactive protein, *DNA* deoxyribonucleic acid, *ESR* erythrocyte sedimentation rate, *ELA-2* elastase 2, *IL-6* interleukin-6, *LF* lactoferrin, *LR+* positive likelihood ratio, *LR−* negative likelihood ratio, *NETs* neutrophil extracellular traps, *NPV* negative predictive value, *PMN%* polymorphonuclear neutrophil percentage, *PCR* polymerase chain reaction, *PPV* positive predictive value, *sICAM-1* soluble intercellular adhesion molecule-1, *TLR2* toll-like receptor 2, *TNF-alpha* tumor necrosis factor-alpha, *WBC* white blood cell count

### Literature search

Electronic search strategies will be developed and tested by an experienced medical information specialist in consultation with the review team (please see search strategy in Additional file 2) [27]. Using the OVID platform, we will search Ovid MEDLINE®, MEDLINE® In-Process & Other Non-Indexed Citations, and Embase. Using the Cochrane Library on Wiley (including Cochrane), we will search the following: Database of Systematic Reviews, DARE, CENTRAL, HTA, and NHS EED. Vocabulary and syntax will be adjusted across databases.

Strategies will utilize a combination of controlled vocabulary (e.g., “prosthesis-related infections,” “joint prosthesis/adverse effects,” “arthritis, infectious/diagnosis”) and keywords (e.g., “periprosthetic joint infection,” “replacement joint infection,” “PJI”). When possible, animal-only studies and opinion pieces will be removed.

We will perform a search for gray literature using CADTH’s Grey Matters Light [28]. Additional references will be sought through hand-searching the bibliographies of relevant studies.

### Study screening and selection

Screening will be performed by uploading citations into an online systematic review software program (Distiller Systematic Review (DSR) Software©) [29] and will involve a two-step process:

- Step 1: title/abstract screening
- Step 2: full-text screening

At both levels, eligibility will be determined independently by two reviewers. Initial piloting will involve 20–25 citations as training dataset to reach acceptable levels of agreement between the reviewers.

Disagreements among reviewers will be resolved through consensus or third party adjudication. Reports that are duplicates or co-publications of studies will be identified. Following full-text screening, a list of excluded studies with reasons for exclusion will be provided in an appendix of the final report.

We will begin with screening published and unpublished records and select those that meet inclusion/exclusion criteria. Our search of literature will involve both primary studies and systematic reviews. The latter will be used as an additional source of primary studies.

It is understood that the full literature search may uncover novel biomarkers and these will be included if the studies meet the other criteria.

### Data extraction

We will develop data extraction forms and pilot test on a sample of studies to achieve acceptable levels of agreement between the two data extractors. At a minimum, the following information will be extracted with additional data elements being added as deemed appropriate during the review process:

- *Study characteristics*: author, year of publication, country, design, sample size, clinical setting, joints affected, duration of follow-up, number studied (or randomized in the case of RCTs) and number analyzed for each outcome, number of drop-outs with reason, and funding source.
- *Population characteristics*: inclusion/exclusion criteria; patient characteristics such as mean age, race, sex, BMI, and history of joint arthroplasty or previous surgery; surgical-related characteristics such as surgeon and hospital volume, joint (knee, hip, shoulder), operative time, previous procedure in operating room, anesthetic management, postoperative risk factors prior to discharge (e.g., persistent postoperative wound drainage, distant infection, and length of stay), postdischarge (e.g., dental work, subsequent surgery), and time since prosthesis implantation.
- *Intervention characteristics*: timing of sampling; method of sampling (e.g., location of swab), method of measuring, threshold, frequency, and subsequent management.
- *Gold standard*: To the best of our knowledge, there is no generally accepted gold standard, so we will be guided by those used in published studies. These seem to include simple standard culture of joint aspirate, prolonged culture of sonicated biofilm or explanted prosthesis, and final adjudication of cases by an expert panel after full follow-up. We will work with our knowledge users (orthopedic surgeons and infectious disease specialists) to categorize the likely accuracy of the gold standards used by investigators and determine what impact these have on measures of diagnostic test performance.
- *Outcomes*: definitions of outcomes of review interest, outcomes data (e.g., false/true positive, false/true negative from 2 × 2 table for diagnostic studies), sensitivity and specificity, negative predictive value (NPV) and positive predictive value (PPV), and positive likelihood ratio (LR+) and negative likelihood ratio (LR−). Data will be extracted by a single reviewer with all outcomes data verified by a second reviewer. For each test/marker, information will be summarized and presented in an evidence table and summary of findings table.

### Quality appraisal

We will assess the quality of the included studies of diagnostic test accuracy using the QUADAS-2 tool [29]. Risk of bias assessments will be done by one reviewer, with another reviewer providing verification to all of the assessed studies. Based on the QUADAS-2 guidance, we will tailor the tool according to our research question (please see Additional file 3 and Additional file 4 for further details).

### The Grading of Recommendations Assessment, Development and Evaluation (GRADE)

If we find a sufficiently comprehensive literature for some of the candidate tests, we will attempt to apply the GRADE methodology to rate the quality of the body of evidence as high, moderate, low, or very low [30]. Although GRADE may have not been extensively used in diagnostic accuracy reviews and the Cochrane collaboration may have not yet officially recommended its use for such reviews, we will attempt piloting incorporation of GRADE in this review. We will create a summary of evidence table using the Grade Development Tool [31]. To arrive at a rating, two reviewers will independently assess the body of evidence for each gradable outcome according to risk of bias, consistency, directness, precision, and study design [32, 33]. Quality of evidence will be judged for estimates of test performance (true positive (TP), false positive (FP), true negative (TN), and false negative (FN)) using previously published GRADE guidance [32].

### Statistical analyses and evidence synthesis

Where data allow, each individual index test will be compared against a reference test (gold standard—see above). For each marker/test, TP, TN, FP, and FN will be retrieved. Where authors do not report those values but provide the raw data, we will calculate the values considering high sensitivity (95 %) for prioritized blood markers, and high specificity (95 %) for prioritized synovial and tissue tests. An evidence summary table will be reported and each study will be presented in a forest plot. The forest plot will display the data from the sensitivity and specificity values of the marker test and the corresponding 95 % confidence intervals. Heterogeneity that may be explained by clinical or methodological differences between studies may preclude meta-analyses, as will general sparsity of data or high risk of bias affecting most or all of the relevant studies. If studies use a common threshold, then we will estimate summary sensitivity and specificity.

For such analysis, we will use the bivariate model (Reitsma [34]) which models the correlation between sensitivity and specificity directly and assumes random effects to account for the between study heterogeneity. We will present the results in a summary receiver operating characteristic (ROC) curve, summary sensitivity and specificity, 95 % confidence region around the summary estimates, and 95 % confidence region.

If there is evidence of threshold effect, then we will estimate summary ROC curves using the hierarchical SROC (HSROC) model of Rutter and Gatsonis (Rutter [35]) which will allow study level covariates to be added in the model. In such cases, we will present the results in a summary ROC curve. From the summary ROC curve, the expected sensitivity at a given value of specificity (or vice versa) can be computed [34]. We will compare index tests against each other using one of the two approaches (depending on the available data) suggested by the *Cochrane Handbook for Diagnostic Test Accuracy Reviews* [34].

Both the bivariate model and HSROC model can be used to investigate the relative accuracy of two index tests depending on the nature of the available data (common or variable threshold). We will run both the hierarchical models in SAS software according to the methods of Macaskill et al. as described in the *Cochrane Handbook for Diagnostic Test Accuracy Reviews* [34]. If the data allow, we will perform sensitivity analysis based on risk of bias (e.g., treating “unclear risk of bias” as “low risk” and “high risk,” and removing “high risk of bias” studies from the analyses) or other PICO elements (previous procedure in operating room, anesthetic management, postoperative risk factors prior to discharge, and postdischarge factors, e.g., dental work). We will conduct subgroup analyses for the following subgroups:

1. Patients undergoing primary arthroplasty
2. Patients undergoing revision arthroplasty
3. Various gold standards used in the included studies
4. Study design, e.g., randomized controlled trials, observational studies
5. Specific affected joint: shoulder, hip, and knee
6. Time since prosthesis implantation: acute versus chronic PJI
7. Reasons for arthroplasty, e.g., septic and aseptic

## Discussion

Total replacements for the hip, knee, and shoulder joints have proven to be highly successful and cost-effective for alleviating pain and improving function in patients with disabling joint disease. However, developing a periprosthetic joint infection (PJI) after a total joint replacement (TJR) is currently one of the most serious and challenging complications that face our patients and their families, clinicians, and our healthcare system at large. Diagnosing PJI has remained challenging due to the lack of a gold standard, which makes it difficult to diagnose and treat patients in a timely fashion. This affects patients’ quality of life, enhances suffering, results in poor outcomes, and increases the economic burden on the healthcare system. None of the existing clinical guidelines on diagnosing PJI is based on a systematic review of all available evidence [2–5].

In 2014, over 100,000 primary hip and knee replacement were performed in Canada according to the Canadian Joint Replacement Registry. This translates into a 5-year increase of over 15 %. The annual incidence increase is estimated to be over 200 cases, most due to Canada’s growing geriatric population [36]. Considering this prediction, we need to urgently develop strategies in order to establish a timely and accurate diagnosis of PJI.

We believe that a higher quality of scientific rigor is necessary to establish a diagnostic guideline that represents current evidence more accurately relative to guidelines based on consensus, expert opinions, and narrative reviews. Therefore, we will conduct a systemic review of diagnostic testing (blood markers, synovial fluids, and tissue testing) for PJI using appropriate methodologies and quality assessment tools that may feed into an evidence-based clinical practice guideline.

The limitations of previous clinical guidelines include their being based on consensus, expert opinion, and narrative reviews. Additionally, the use of gold standard has been inconsistent throughout the literature and other guidelines.

The strength of this evidence lies in its status as a systematic literature search, its coverage of three major joints (hip, knee, and shoulder), and the fact that it will be guided by knowledge users (an orthopedist and infectious disease specialists).

We will compare the findings of this review, including the diagnostic performance of various tests, with those provided by other consensus-based guidelines, and we will discuss the differences and similarities in point estimates. This systematic review will constitute a foundation for evidence-based guides on diagnostic performance of blood markers, synovial fluid tests, and tissue cultures; these guides will provide recommendations to clinicians and surgeons for diagnosing PJI accurately and efficiently. Currently, there are no clear clinical pathways to diagnose PJI, and available consensus-based guidelines lack the evidence of indicating how these tests should be triaged and added on or used to replace an existing test. This systematic review can help address this gap and may also identify knowledge gaps in PJI diagnosis that could direct further research in the field.

## Additional files

Additional file 1:
**PRISMA-P checklist.** (DOCX 34 kb) Additional file 2:
**Search strategy for periprosthetic joint infection systematic review.** (DOCX 14 kb) Additional file 3:
**List of risk factors that will determine the risk status of population in an included study.** (DOCX 18 kb) Additional file 4:
**Additional questions and items in QUADAS-2 tool in order to tailor the tool based on our research question.** (DOCX 13 kb)

## Abbreviations

α: alpha
AFB: acid-fast bacillus
Cf-DNA: circulating free DNA
CRP: C-reactive protein
CD64: cluster of differentiation 64
DNA: deoxyribonucleic acid
ESR: erythrocyte sedimentation rate
ELA-2: elastase 2
IL-6: interleukin-6
LF: lactoferrin
LR+: positive likelihood ratio
LR−: negative likelihood ratio
NPV: negative predictive value
NETs: neutrophil extracellular traps
PJI: periprosthetic joint infection
PMN%: polymorphonuclear neutrophil percentage
PCR: polymerase chain reaction
PPV: positive predictive value
sICAM-1: soluble intercellular adhesion molecule-1
TLR2: toll-like receptor 2
TNF-alpha: tumor necrosis factor-alpha
TJR: total joint replacement
WBC: white blood cell count

## References

[1] Shearer DW, Youm J, Bozic KJ (2015). Short-term complications have more effect on cost-effectiveness of THA than implant longevity. Clin Orthop Relat Res.

[2] Parvizi J, Zmistowski B, Berbari EF, Bauer TW, Springer BD, Della Valle CJ (2011). New definition for periprosthetic joint infection: from the Workgroup of the Musculoskeletal Infection Society. Clin Orthop Relat Res.

[3] Segawa H, Tsukayama DT, Kyle RF, Becker DA, Gustilo RB (1999). Infection after total knee arthroplasty. A retrospective study of the treatment of eighty-one infections. J Bone Joint Surg Am.

[4] Cats-Baril W, Gehrke T, Huff K, Kendoff D, Maltenfort M, Parvizi J (2013). International consensus on periprosthetic joint infection: description of the consensus process. Clin Orthop Relat Res.

[5] Kapadia BH, Berg RA, Daley JA, Fritz J, Bhave A, Mont MA. Periprosthetic joint infection. Lancet. 2015. doi: 10.1016/S0140-6736(14)61798-0 10.1016/S0140-6736(14)61798-0 26135702

[6] Matar WY, Jafari SM, Restrepo C, Austin M, Purtill JJ, Parvizi J (2010). Preventing infection in total joint arthroplasty. J Bone Joint Surg Am.

[7] Bozic KJ (2015). The role of the payment system in improving value in healthcare. J Arthroplasty.

[8] Kurtz SM, Lau E, Watson H, Schmier JK, Parvizi J (2012). Economic burden of periprosthetic joint infection in the United States. J Arthroplasty.

[9] Parvizi J (2015). Periprosthetic joint infection: the last frontier. Bone Joint J.

[10] George DA, Gant V, Haddad FS (2015). The management of periprosthetic infections in the future: a review of new forms of treatment. Bone Joint J.

[11] Saltzman MD, Marecek GS, Edwards SL, Kalainov DM (2011). Infection after shoulder surgery. J Am Acad Orthop Surg.

[12] Del Arco A, Bertrand ML (2013). The diagnosis of periprosthetic infection. Open Orthop J.

[13] Topolski MS, Chin PY, Sperling JW, Cofield RH (2006). Revision shoulder arthroplasty with positive intraoperative cultures: the value of preoperative studies and intraoperative histology. J Shoulder Elbow Surg.

[14] Berbari E, Mabry T, Tsaras G, Spangehl M, Erwin PJ, Murad MH (2010). Inflammatory blood laboratory levels as markers of prosthetic joint infection: a systematic review and meta-analysis. J Bone Joint Surg Am.

[15] Font-Vizcarra L, Garcia S, Martinez-Pastor JC, Sierra JM, Soriano A (2010). Blood culture flasks for culturing synovial fluid in prosthetic joint infections. Clin Orthop Relat Res.

[16] Fink B, Gebhard A, Fuerst M, Berger I, Schafer P (2013). High diagnostic value of synovial biopsy in periprosthetic joint infection of the hip. Clin Orthop Relat Res.

[17] Fink B, Makowiak C, Fuerst M, Berger I, Schafer P, Frommelt L (2008). The value of synovial biopsy, joint aspiration and C-reactive protein in the diagnosis of late peri-prosthetic infection of total knee replacements. J Bone Joint Surg (Br).

[18] Tsaras G, Maduka-Ezeh A, Inwards CY, Mabry T, Erwin PJ, Murad MH (2012). Utility of intraoperative frozen section histopathology in the diagnosis of periprosthetic joint infection: a systematic review and meta-analysis. J Bone Joint Surg Am.

[19] Gallo J, Kolar M, Dendis M, Loveckova Y, Sauer P, Zapletalova J (2008). Culture and PCR analysis of joint fluid in the diagnosis of prosthetic joint infection. New Microbiol.

[20] Gallo J, Raska M, Dendis M, Florschutz AV, Kolar M (2004). Molecular diagnosis of prosthetic joint infection. A review of evidence. Biomed Pap Med Fac Univ Palacky Olomouc Czech Repub.

[21] Parvizi J, Huang R, Raphael IJ, Maltenfort MG, Arnold WV, Rothman RH (2015). Timing of symptomatic pulmonary embolism with warfarin following arthroplasty. J Arthroplasty.

[22] Gehrke T, Kendoff D (2012). Peri-prosthetic hip infections: in favour of one-stage. Hip Int.

[23] Diagnostic Test Accuracy Working Group Cochrane diagnostic test accuracy reviews. [Internet]. [http://dta.cochrane.org/handbook-dta-reviews]. Accessed: 13-10-2015.

[24] Moher D, Liberati A, Tetzlaff J, Altman DG (2009). Preferred reporting items for systematic reviews and meta-analyses: the PRISMA statement. J Clin Epidemiol.

[25] Shamseer L, Moher D, Clarke M, Ghersi D, Liberati A, Petticrew M (2015). Preferred reporting items for systematic review and meta-analysis protocols (PRISMA-P) 2015: elaboration and explanation. BMJ.

[26] Booth A, Clarke M, Dooley G, Ghersi D, Moher D, Petticrew M (2012). The nuts and bolts of PROSPERO: an international prospective register of systematic reviews. Syst Rev.

[27] Sampson M, McGowan J, Cogo E, Grimshaw J, Moher D, Lefebvre C (2009). An evidence-based practice guideline for the peer review of electronic search strategies. J Clin Epidemiol.

[28] Canadian Agency for Drugs and Technologies in Health CADTH. Grey matters light. [Internet]. [https://www.cadth.ca/sites/default/files/is/cadth_Handout_greymatters_light_e.pdf]. Accessed: 13-10-2015.

[29] Evidence Partners DistillerSR [Computer Program]. [http://systematic-review.net/]. Accessed: 13-10-2015.

[30] Guyatt GH, Oxman AD, Vist GE, Kunz R, Falck-Ytter Y, Alonso-Coello P (2008). GRADE: an emerging consensus on rating quality of evidence and strength of recommendations. BMJ.

[31] Evidence Prime GRADEpro. Guideline Development Tool (GDT). [Internet]. [http://www.guidelinedevelopment.org/]. Accessed: 13-10-2015.

[32] Schunemann HJ, Oxman AD, Brozek J, Glasziou P, Jaeschke R, Vist GE (2008). Grading quality of evidence and strength of recommendations for diagnostic tests and strategies. BMJ.

[33] Singh S, Chang SM, Matchar DB, Bass EB (2012). Chapter 7: grading a body of evidence on diagnostic tests. J Gen Intern Med.

[34] Reitsma JB, Glas AS, Rutjes AW, Scholten RJ, Bossuyt PM, Zwinderman AH (2005). Bivariate analysis of sensitivity and specificity produces informative summary measures in diagnostic reviews. J Clin Epidemiol.

[35] Rutter CM, Gatsonis CA (2001). A hierarchical regression approach to meta-analysis of diagnostic test accuracy evaluations. Stat Med.

[36] Canadian Institute for Health Information. Increase in hip and knee replacements over past 5 years. [Internet]. [http://www.cihi.ca/web/resource/en/cjrr2014_publicsummary_en.pdf]. Accessed: 13-10-2015.

[37] Lijmer JG, Mol BW, Heisterkamp S, Bonsel GJ, Prins MH, van der Meulen JH (1999). Empirical evidence of design-related bias in studies of diagnostic tests. JAMA.

[38] Deeks J, Egger M, Smith G, Altman D (2001). Systematic reviews of evaluations of diagnostic and screening tests. Systematic reviews in health care: meta-analysis in context.

[39] Bruening W, Uhl S, Fontanarosa J, Reston J, Treadwell J, Schoelles K. Noninvasive diagnostic tests for breast abnormalities: update of a 2006 review. [Internet]. [http://www.ncbi.nlm.nih.gov/pubmedhealth/n/cer47/pdf/]. Accessed: 13-10-2015. 22420009

