# Erratum to: A protocol for a systematic review of the diagnostic accuracy of blood markers, synovial fluid, and tissue testing in periprosthetic joint infections (PJI)

**DOI:** 10.1186/s13643-016-0281-x

**Published:** 2016-06-20

**Authors:** Paul E. Beaule, Beverley Shea, Hesham Abdelbary, Nadera Ahmadzai, Becky Skidmore, Ranjeeta Mallick, Brian Hutton, Alexandra C. Bunting, Julian Moran, Roxanne Ward, David Moher

**Affiliations:** Division of Orthopaedic Surgery, University of Ottawa, 501 Smyth Rd., Ottawa, ON K1H 8L6 Canada; Knowledge Synthesis Group, Ottawa Hospital Research Institute, Center for Practice-Changing Research, 501 Smyth Rd., Ottawa, ON K1H 8L6 Canada; Ottawa Hospital Research Institute, 501 Smyth Rd., Ottawa, ON K1H 8L6 Canada; University of Ottawa Faculty of Medicine, 451 Smyth Rd., Ottawa, ON K1H 8L1 Canada; Bruyère Research Institute, 85 Primrose Ave., Ottawa, ON K1R 6M1 Canada; School of Epidemiology, Public Health and Preventive Medicine, Faculty of Medicine, University of Ottawa, Ottawa, ON Canada

## Erratum

Following publication, it was noticed that there was a reference missing in this article [1]. Regarding the italicised text below from section *Statistical analyses and evidence synthesis* of the original article:

*We will compare index tests against each other using one of the two approaches (depending on the available data) suggested by the Cochrane Handbook for Diagnostic Test Accuracy Reviews [34].*

*Both the bivariate model and HSROC model can be used to investigate the relative accuracy of two index tests depending on the nature of the available data (common or variable threshold). We will run both the hierarchical models in SAS software according to the methods of Macaskill et al. as described in the Cochrane Handbook for Diagnostic Test Accuracy Reviews [34].*

Reference number 34 should in fact be the reference below:

Macaskill P, Gatsonis C, Deeks JJ, Harbord RM, Takwoingi Y. Chapter 10: Analysing and Presenting Results. In: Deeks JJ, Bossuyt PM, Gatsonis C (editors), Cochrane Handbook for Systematic Reviews of Diagnostic Test Accuracy Version 1.0. The Cochrane Collaboration, 2010. Available from: http://srdta.cochrane.org/.

Additionally, Dr Abdelbary’s surname included a typographical error. The correct spelling is: Abdelbary. The updated author list is therefore as follows:

Paul E. Beaule, Beverley Shea, Hesham Abdelbary, Nadera Ahmadzai, Becky Skidmore, Ranjeeta Mallick, Brian Hutton, Alexandra C. Bunting, Julian Moran, Roxanne Ward and David Moher.
