# Supplementary material for: A protocol for a systematic review of the diagnostic accuracy of blood markers, synovial fluid, and tissue testing in periprosthetic joint infections (PJI)
Source: Syst Rev. 2015 Nov 2;4:148. doi: 10.1186/s13643-015-0124-1 (PMC4630899; doi:10.1186/s13643-015-0124-1)
Supplement: Additional file 1: — PRISMA-P checklist. (DOCX 34 kb) [file 13643_2015_124_MOESM1_ESM.docx]

**Additional file 1:** PRISMA P checklist

| **PRISMA-P 2015 checklist: recommended items to include in a systematic review protocol** | | |
| --- | --- | --- |
| **Section/topic** | **Item #** | **Checklist item** |
| **ADMINISTRATIVE INFORMATION** | | |
| **Title** | | |
| **Identification** | 1a | A Protocol for a Systematic Review of the Diagnostic Accuracy of Blood Markers, Synovial fluid and Tissue Testing in Peri-prosthetic Joint Infections (PJI) |
| **Update** | 1b | Not applicable |
| **Registration** | 2 | This review is registered with PROSPERO (registration number: CRD42015023768). |
| **Authors** | | |
| **Contact** | 3a | Authors:  Paul E. Beaule, Professor, MD, FRCSC^1^; Beverley Shea, PhD^2^; Hesham Abedlbary, Assistant Professor, MD^1^; Nadera Ahmadzai, MD, MPH, MSc^2^; Becky Skidmore, MLS^2^; Brian Hutton, PhD^2^; Alexandra C. Bunting, BSc^3^; Julian Moran^4^; Roxanne Ward, RN, BA, MSc^2^; David Moher, PhD^2^  Institutional Affiliations:  ^1^ Division of Orthopaedic Surgery, University of Ottawa, 501 Smyth Rd. Ottawa, ON, K1H 8L6, Canada  ^2^ Knowledge Synthesis Group, Ottawa Hospital Research Institute, 501 Smyth Rd. Ottawa, ON, K1H 8L6, Canada  ^3^ University of Ottawa Faculty of Medicine, 451 Smyth Rd. Ottawa, ON, K1H 8L1 Canada  ^4^ Bruyère Research Institute, 85 Primrose Ave. Ottawa, ON, K1R 6M1 Canada  Email addresses:  Paul E. Beaule: [pbeaule@toh.on.ca](mailto:pbeaule@toh.on.ca)  Beverley Shea: [bevshea35@gmail.com](mailto:bevshea35@gmail.com)  Hesham Abedlbary: [habdelbary@toh.on.ca](mailto:habdelbary@toh.on.ca)  Nadera Ahmadzai: [nahmadzai@ohri.ca](mailto:nahmadzai@ohri.ca)  Becky Skidmore: [bskidmore@rogers.com](mailto:bskidmore@rogers.com)  Brian Hutton: [bhiddy9999@gmail.com](mailto:bhiddy9999@gmail.com)  Alexandra C. Bunting: [alexandra.bunting@uottawa.ca](mailto:alexandra.bunting@uottawa.ca)  Julian Moran: [juliandeanmoran@gmail.com](mailto:juliandeanmoran@gmail.com)  Roxanne Ward: [rward@ohri.ca](mailto:rward@ohri.ca)  David Moher: [dmoher@ohri.ca](mailto:dmoher@ohri.ca)  Corresponding author:  Nadera Ahmadzai, MD, MPH, MSc  Ottawa Hospital Research Institute  Center for Practice-changing Research  501 Smyth Rd Ottawa, ON, K1H 8L6  Canada  Email: [nahmadzai@ohri.ca](mailto:nahmadzai@ohri.ca) |
| **Contributions** | 3b | PB, BS, DM, HA, BS, and NA were involved in conception and design of this review protocol. They may be also be involved in interpreting data, grading evidence, constructing, the overall results and for discussion and contributing significant intellectual and clinical content. BH will conduct the statistical analyses. AB, JM, RW, and NA will be involved in one or more than one of the following tasks: data extraction, verification, and quality appraisal of evidence. NA will design and implement the data extraction form, prepare and process data for analyses; analyze, organize and interpret data; synthesize the evidence and prepare the first draft of the review. All authors will read and approve the final version of the manuscript.  Guarantor: NA |
| **Amendments** | 4 | Any amendments will be explained in the final manuscript of the review. |
| **Support** | | |
| **Sources** | 5a |  |
| **Sponsor** | 5b | We have applied for CIHR funding |
| **Role of sponsor/funder** | 5c | Not applicable now |
| **INTRODUCTION** | | |
| **Rationale** | 6 | Total joint replacement (TJR) procedures have been one of the most rewarding interventions to treat patients suffering from joint disease. However, developing a periprosthetic joint infection (PJI) is a serious complication that is associated with the highest burden of cost and reduction in patients’ quality of life when compared to other complications after TJRs. One of the main challenges facing clinicians who are treating PJIs is confirming the accurate diagnosis of infection in a timely fashion. Multiple orthopaedic associations have published clinical guidelines for diagnosing PJI which are solely based on consensus approaches, expert opinions and narrative reviews as well as not covering all types of prosthetic joints. We believe that a higher quality of scientific rigour is necessary to establish a diagnostic guideline that translates current evidence more accurately, and that identifies important knowledge gaps in methods of diagnosing PJI. Therefore, we would like to conduct a systematic review on diagnostic performance of blood markers, synovial fluids, and tissue tests for diagnosing PJI. |
| **Objectives** | 7 | Research Question: What is the current evidence for the accurate diagnosis of PJIs using published protocols that support the use of blood markers, synovial fluid and tissue testing as diagnostic tools compared to gold standard in patients undergone joint replacement of knee, hip and shoulder. The outcomes of interest are diagnostic point estimates of the individual index tests (sensitivity, specificity, Likelihood ratio positive, likelihood ratio negative, negative predictive values and positive predictive values). |
| **METHODS** | | |
| **Eligibility criteria** | 8 | **Population:** Patients’ who have undergone joint replacement of knee, hip and shoulder (no time frame as infections may be chronic)  **Index tests:**  blood markers, synovial tests, and tissue culture tests:  Blood markers:   - Serum c-reactive protein (CRP) - Serum erythrocyte sedimentation rate (ESR) - Serum white blood cell (WBC) count - Serum interleukin-6 (IL-6) levels - Serum procalcitonin levels - Serum interferon-alpha levels - Serum toll-like receptor 2 (TLR2) - Human beta-defensin-3 levels - Neutrophil CD64 - Soluble intercellular adhesion molecule-1 (sICAM-1) - Tumor Necrosis Factor-alpha (TNF-alpha) - α-defensin - neutrophil elastase 2 (ELA-2) - bactericidal/permeability-increasing protein - neutrophil gelatinase-associated lipocalin - Lactoferrin (LF)   Synovial fluid tests:   - Synovial fluid white blood cell (WBC) count - Synovial fluid polymorphonuclear neutrophil percentage (PMN%) analysis - Synovial c-reactive protein (CRP) levels - Synovial neutrophil-derived circulating free DNA/neutrophil extracellular traps (cf-DNA/NETS) - Intra-articular purulence - Application of synovial fluid to leukocyte esterase test strip   Tissue tests:   - Periprosthetic tissue culture/swab culture/joint aspiration culture - Blood culture - Histological analysis: PMN per high power field. - Gram stain - Routine acid-fast bacillus (AFB) testing and fungal testing - Polymerase chain reaction (PCR) and molecular techniques   **Reference test:**  Joint fluid (with cell count, Gram stain, and culture) or tissue (with histopathology and culture). Note: given that there is no gold standard for diagnosing PJI, we may also consider any gold standard used in the primary studies.  **Outcomes:**   - Sensitivity and specificity of individual tests and - Negative predictive value (NPV) and positive predictive value (PPV) - Positive likelihood ratio (LR+) and negative likelihood ratio (LR-)   **Study designs:**  We will include randomized controlled trials, cross-sectional, systematic reviews, controlled cohort and case-control studies (recognizing that the latter may over-estimate test performance)  **Exclusion:** Case reports, commentaries, expert opinion, narrative reviews, Studies that are not on diagnostic performance of the index tests, Patients who undergone joint replacements of other joints; and Animal studies, and imaging indexttests |
| **Information sources** | 9 | Please see item # 10 below. |
| **Search strategy** | 10 | Electronic search strategies will be developed and tested by an experienced medical Information Specialist in consultation with the review team (please see search strategy in additional file 1). The searches will be done using the OVID platform to include MEDLINE®, MEDLINE® In-Process & Other Non-Indexed Citations and EMBASE. The Cochrane Library on Wiley (including Cochrane Database of Systematic Reviews, DARE, CENTRAL, HTA, and NHS EED) will also be included in the search.  We will perform a search for grey literature using the checklist from CADTH’s Grey Matters Light. Strategies will utilize a combination of controlled vocabulary (e.g., prosthesis-related infections, joint prosthesis/adverse effects, arthritis, infectious/diagnosis) and keywords (e.g., periprosthetic joint infection, replacement joint infection, PJI). Vocabulary and syntax will be adjusted across databases. Additional references will be sought for through hand-searching the bibliographies of relevant studies. |
| **Study records** | | |
| **Data management** | 11a | Screening will be done by uploading citations into an online systematic review software (Distiller Systematic Review (DSR) Software©) and data will be stored there. |
| **Selection process** | 11b | Two reviewers will independently screen the literature for inclusion using the prespecified eligibility criteria. Screening will involve a two-step process, namely;   - Level 1: Involves the title/abstract screening - Level 2: Involves full-text screening   At both levels, eligibility will be determined by two reviewers. Initial piloting will involve 20-25 citations as training data-set to reach acceptable levels of agreement between the reviewers. |
| **Data collection process** | 11c | Data extraction forms will be developed and pilot tested on a sample of studies to achieve acceptable levels of agreement between the two data extractors.  Data extractions by reviewers will be verified and disagreements will be resolved through consensus or third party adjudication. |
| **Data items** | 12 | The following data items in addition will be extracted:   - Study Characteristics: author; year of publication; country; design; sample size; clinical setting; joints affected, duration of follow-up; number studied (or randomized in the case of RCTs) and number analyzed for each outcome; number of drop-outs with reason; and funding source. - Population Characteristics: inclusion/exclusion criteria; patient characteristics such as mean age, race, BMI, history of joint arthroplasty or previous surgery, presence of malignancy, system surgical patient risk index score, socioeconomic status (SES) status, malnutrition, morbid obesity (BMI >40 kg/m2), previous prosthetic joint infection, revision arthroplasty, revision arthroplasty,co-morbidities [e.g. hypercalcemia with or without diabetes, poorly controlled diabetes malitus (glucose N200 mg/L orHbA1 >N7%), systematic malignancy, human immunodeficiency virus (HIV), sickle cell hemoglobinopathies, hemophilia, malnutrition, posttraumatic arthritis], medications (e.g. non-steroidal anti-inflammatory drugs (NSAIDs), platelet function inhibitors (e.g. clopidogrel, low dose aspirin), anticoagulants (e.g. warfarin, heparin), previous operation in the same joint, place of residence, indices of comorbidities [e.g. American Society of Anesthesiologists (ASA) score, Charlson Index, National Nosocomial Infections Surveillance (NNIS) System surgical patient risk index], rheumatoid arthritis, active infection of the arthritic joint (septic arthritis), presence of septicemia, and/or presence of active local cutaneous, subcutaneous, or deep tissue infection, active liver disease, chronic renal disease, excessive smoking (>one pack per day), excessive alcohol consumption (>40 units per week), intravenous drug abuse, recent hospitalization, extended stay in a rehabilitation facility, male gender, diagnosis of post-traumatic arthritis, inflammatory arthropathy, prior surgical procedure in the affected joint, and severe immunodeficiency, and preoperative diagnosis of posttraumatic arthritis with or without prior surgery. Surgical –related characteristics such as surgeon and hospital volume, joint (knee, hip, shoulder), operative time, previous procedure in operating room, anesthetic management, post operative risk factors prior to discharge (e.g. persistent postoperative wound drainage, surgical wound-related complications, distant infection, cardiovascular complications, allogenic blood transfusion, and length of stay), post discharge (e.g. dental work, subsequent surgery), and long-term stay in healthcare facility. - Intervention Characteristics timing of sampling; method of sampling (e.g. location of swab), method of measuring, expertise of personnel administering test; and any details about quality assurance processes used (the same information will be collected for comparator tests where more than one are being compared). - Gold Standard: there is no generally accepted gold standard, so we will be guided by those used in published studies. These seem to include simple standard culture of joint aspirate, prolonged culture of sonicated biofilm or explanted prosthesis, and final adjudication of cases by an expert panel after full follow-up. We will work with our knowledge users (orthopedic surgeons and infectious disease specialists) to categorize the likely accuracy of the gold standards used by investigators and determine what impact these have on measures of diagnostic test performance. - Outcomes: Please see item # 13 below. |
| **Outcomes and prioritization** | 13 | Outcomes: outcomes data (e.g. false/true positive, false/true negatives from 2x2 table for diagnostic studies; sensitivity and specificity; negative predictive value (NPV) and positive predictive value (PPV); positive likelihood ratio (LR+) and negative likelihood ratio (LR-) |
| **Risk of bias in individual studies** | 14 | We will assess the quality of individual studies of diagnostic test accuracy using the QUADAS-2 tool. All risk of bias assessments will be done by one reviewer, with another reviewer providing verification. We will consider the risk of bias assessment in synthesizing the results.  If we find a sufficiently comprehensive literature for some of the candidate tests we will attempt to apply the GRADE methodology to rate the quality of the body of evidence as high, moderate, low, or very low. We will create a Summary of Evidence table using the Grade Development Tool. To arrive at a rating, two reviewers will independently assess the body of evidence for each gradable outcome according to risk of bias, consistency, directness, precision, publication bias and study design. Quality of evidence will be judged for estimates of test performance (True Positive (TP), False Positive (FP), True Negative (TN), and False Negative (FN)) using previously published GRADE guidance. |
| **Data** | | |
| **Synthesis** | 15a | Heterogeneity that may be explained by clinical or methodological differences between studies may preclude meta-analyses, as will general sparsity of data or high risk of bias affecting most or all of the relevant studies. |
|  | 15b | Where data allow, each individual index test will be compared against a reference test. For each marker/test, TP, TN, FP, and FN will be retrieved. An evidence summary table will be reported and each study will be presented in a forest plot. The forest plot will display the data from the sensitivity and specificity values of the marker test, and the corresponding 95% confidence intervals.  If studies use a common threshold and estimates of diagnostic accuracy are consistent across studies (e.g. sensitivity, specificity) as evident from a summary Receiver Operating Characteristic (sROC) curve, then we will pool sensitivities and specificities separately and present them as coupled estimates. If there is evidence of an implicit or explicit threshold effect, then we will pool the data using the hierarchical bivariate random-effects model for calculating summary estimates of sensitivity and specificity, with 95% confidence intervals . For pooled analyses we will present coupled sensitivity and specificity plots, summary receiver operating curves (sROC) with 95% confidence intervals and prediction region, and average (i.e. pooled) sensitivity, specificity, LR+, and LR- with 95% confidence intervals. All sROC plots will present study level estimates using points sized according to study sample size. |
|  | 15c | If the data allow we will perform sensitivity analysis based on risk of bias or other PICO elements, study design characteristics and the gold standards used by the authors. |
|  | 15d | If studies are not pooled, we will present coupled sensitivity and specificity plots and synthesizing evidence qualitatively. |
| **Meta-bias(es)** | 16 | Not planned. |
| **Confidence in cumulative evidence** |  | We will use GRADE to grade the strength of body of evidence. |
