# Supplementary material for: A protocol for a systematic review of the diagnostic accuracy of blood markers, synovial fluid, and tissue testing in periprosthetic joint infections (PJI)
Source: Syst Rev. 2015 Nov 2;4:148. doi: 10.1186/s13643-015-0124-1 (PMC4630899; doi:10.1186/s13643-015-0124-1)
Supplement: Additional file 2: — Search strategy for periprosthetic joint infection systematic review. (DOCX 14 kb) [file 13643_2015_124_MOESM2_ESM.docx]

**Additional file 2**

**Appendix 2:** Search Strategy for Periprosthetic Joint Infection systematic review

OVID Searches

Database: Ovid MEDLINE(R) In-Process & Other Non-Indexed Citations and Ovid MEDLINE(R) <1946 to Present>, Embase <1980 to 2014 Week 20>

Search Strategy:

--------------------------------------------------------------------------------

1 Prosthesis-Related Infections/ (204609)

2 exp Joint Prosthesis/ae and (infect* or sepsis or septic*).tw. (2095)

3 ((periprosthetic* or peri-prosthetic*) adj3 (infect* or sepsis or septic)).tw. (1639)

4 (PJI or PJIs).tw. (530)

5 (((prosthe* or periprosthetic* or peri-prosthetic*) adj3 (joint$1 or knee$1 or hip or hips or shoulder$1 or wrist$1 or ankle$1 or elbow$1)) and (infect* or sepsis or septic)).tw. (5835)

6 (((arthroplas* or replacement*) adj3 (joint$1 or knee$1 or hip or hips or shoulder$1 or wrist$1 or ankle$1 or elbow$1)) and (infect* or sepsis or septic)).tw. (14054)

7 ((replacement* adj3 arthroplas*) and (infect* or sepsis or septic)).tw. (226)

8 or/1-7 (218235)

9 Prosthesis-Related Infections/di [Diagnosis] (6560)

10 exp Sepsis/di [Diagnosis] (25516)

11 exp Arthritis, Infectious/di (11528)

12 exp Diagnosis/ (10827394)

13 diagnos*.tw. (3727172)

14 or/9-13 (12471762)

15 8 and 14 (71053)

16 exp Animals/ not (exp Animals/ and Humans/) (7895901)

17 15 not 16 (69357)

18 (comment or editorial or interview or letter or news).pt. (2783837)

19 17 not 18 (67823)

20 limit 19 to yr="2004-current" (52769)

21 20 use prmz (4597) [MEDLINE RECORDS]

22 periprosthetic joint infection/di (93)

23 exp joint prosthesis/ and exp prosthesis infection/ (1429)

24 periprosthetic joint infection/ (309)

25 ((periprosthetic* or peri-prosthetic*) adj3 (infect* or sepsis or septic)).tw. (1639)

26 (PJI or PJIs).tw. (530)

27 (((prosthe* or periprosthetic* or peri-prosthetic*) adj3 (joint$1 or knee$1 or hip or hips or shoulder$1 or wrist$1 or ankle$1 or elbow$1)) and (infect* or sepsis or septic)).tw. (5835)

28 (((arthroplas* or replacement*) adj3 (joint$1 or knee$1 or hip or hips or shoulder$1 or wrist$1 or ankle$1 or elbow$1)) and (infect* or sepsis or septic)).tw. (14054)

29 ((replacement* adj3 arthroplas*) and (infect* or sepsis or septic)).tw. (226)

30 or/23-29 (17962)

31 exp prosthesis infection/di (806)

32 exp Sepsis/di (25516)

33 infectious arthritis/di (3855)

34 exp diagnosis/ (10827394)

35 diagnos*.tw. (3727172)

36 or/31-35 (12467882)

37 30 and 36 (8117)

38 22 or 37 (8117)

39 exp animal experimentation/ or exp models animal/ or exp animal experiment/ or nonhuman/ or exp vertebrate/ (36382875)

40 exp humans/ or exp human experimentation/ or exp human experiment/ (28087060)

41 39 not 40 (8297386)

42 38 not 41 (8032)

43 (letter or editorial).pt. (2474973)

44 42 not 43 (7929)

45 limit 44 to yr="2004-current" (5192)

46 45 use emez (2537) [EMBASE RECORDS]

47 21 or 46 (7134) [MEDLINE & EMBASE RECORDS]

48 limit 47 to yr="2010-current" (3903)

49 remove duplicates from 48 (3110) [DUPLICATES REMOVED, IN BATCHES]

50 47 not 48 (3231)

51 remove duplicates from 50 (2570)

52 49 or 51 (5680) [TOTAL UNIQUE HITS]

53 52 use prmz (4503) [MEDLINE UNIQUE HITS]

54 from 53 keep 1-1000 (1000)

55 from 53 keep 1001-2000 (1000)

56 from 53 keep 2001-3000 (1000)

57 from 53 keep 3001-4000 (1000)

58 from 53 keep 4001-4503 (503)

59 52 use emez (1177) [EMBASE UNIQUE HITS]

60 from 59 keep 1-1000 (1000)

61 from 59 keep 1001-1177 (177)

***************************

Cochrane Library

Search Name: Periprosthetic Joint Infections

Date Run: 19/05/14 13:27:05.74

Description: Final 2014 May 19

ID Search Hits

#1 [mh "Prosthesis-Related Infections"] 172

#2 [mh "Joint Prosthesis"/ae] and (infect* or sepsis or septic):ti,ab,kw 38

#3 ((periprosthetic* or (peri next prosthetic*)) near/3 (infect* or sepsis or septic)):ti,ab,kw 15

#4 (PJI or PJIs):ti,ab,kw 5

#5 (((prosthes* or periprosthetic* or (peri next prosthetic*)) near/3 (joint or joints or knee or knees or hip or hips or shoulder or shoulders or elbow or elbows or wrist or wrists or ankle or ankles)) and (infect* or sepsis or septic)):ti,ab,kw 227

#6 (((arthroplas* or replacement*) near/3 (joint or joints or knee or knees or hip or hips or shoulder or shoulders or elbow or elbows or wrist or wrists or ankle or ankles)) and (infect* or sepsis or septic)):ti,ab,kw 419

#7 ((replacement* near/3 arthroplas*) and (infect* or sepsis or septic)):ti,ab,kw 205

#8 {or #1-#7} 568

#9 [mh "Prosthesis-Related Infections"/di] 17

#10 [mh Sepsis/di] 223

#11 [mh "Arthritis, Infectious"/di] 8

#12 [mh Diagnosis] 253020

#13 diagnos*:ti,ab,kw 51662

#14 {or #9-#13} 282089

#15 #8 and #14 Publication Date from 2004 to 2014 182

DSR - 6

DARE - 28

CENTRAL – 138

NHS EED - 10
