# Supplementary material for: A protocol for a systematic review of the diagnostic accuracy of blood markers, synovial fluid, and tissue testing in periprosthetic joint infections (PJI)
Source: Syst Rev. 2015 Nov 2;4:148. doi: 10.1186/s13643-015-0124-1 (PMC4630899; doi:10.1186/s13643-015-0124-1)
Supplement: Additional file 3: — List of risk factors that will determine the risk status of population in an included study. (DOCX 18 kb) [file 13643_2015_124_MOESM3_ESM.docx]

**Additional File 3:** List of risk factors that will determine the risk status of population in a given study. This risk classification will feed into “patient population” applicability in QUADAS-2 tool.

The followings are considered as risk factors for PJI based on literature and clinical experts opinion:

1. **presence of malignancy**,
2. system surgical patient risk index score,
3. low Socioeconomic status (SES), malnutrition,
4. **morbid obesity (BMI >40 kg/m2),**
5. **previous prosthetic joint infection,**
6. co-morbidities [e.g. hypercalcemia with or without diabetes; **poorly controlled diabetes mellitus (glucose N200 mg/L orHbA1 >N7%); systematic malignancy; human immunodeficiency virus (HIV); sickle cell hemoglobinopathies; hemophilia; malnutrition (albumin< 3);**
7. posttraumatic arthritis, **rheumatoid arthritis, active infection of the arthritic joint (septic arthritis); presence of septicemia, and/or presence of active local cutaneous, subcutaneous, or deep tissue infection; active liver disease; chronic renal disease; excessive smoking (>one pack per day)**; excessive alcohol consumption (>40 units per week); **intravenous drug abuse**; recent hospitalization; male gender; diagnosis of post-traumatic arthritis; **inflammatory arthropathy**; prior surgical procedure in the affected joint; **severe immunodeficiency; preoperative diagnosis of posttraumatic arthritis** with or without prior surgery],
8. **medications** (e.g. Intraarticular **corticosteriod injection** within 3 month prior to surgery, **systemic steroids**, **disease-modifying antirheumatic drugs** **(DMARDs)**, platelet function inhibitors (such as clopidogrel, low dose aspirin),
9. **anticoagulants** (e.g. warfarin, heparin),
10. **previous operation in the same joint**, place of residence,
11. Indices of comorbidities [e.g. American Society of Anesthesiologists (ASA) score, **Charlson Index, National Nosocomial Infections Surveillance (NNIS) System surgical patient** risk index]

The bold font indicates the risk factors that weigh twice the other risk factors. Reviewers will be requested to classify the risk status of a given study population into the followings based on the number of risk factors, proportion of population with the risk factors, and type of risk factors (bolded versus not bolded) in a given study population:

1. Low
2. Moderate
3. High
