# Supplementary material for: A protocol for a systematic review of the diagnostic accuracy of blood markers, synovial fluid, and tissue testing in periprosthetic joint infections (PJI)
Source: Syst Rev. 2015 Nov 2;4:148. doi: 10.1186/s13643-015-0124-1 (PMC4630899; doi:10.1186/s13643-015-0124-1)
Supplement: Additional file 4: — Additional questions and items in QUADAS-2 tool in order to tailor the tool based on our research question. (DOCX 13 kb) [file 13643_2015_124_MOESM4_ESM.docx]

**Additional file 4:** Additional questions/ items in QUADAS 2 tool in order to tailor the tool based on our research question:

Specifically, we will consider the followings:

1. **Patient Selection:** for population applicability, we consider a list of risk factors (please see additional file 3) that will classify the studies’ population risk status into low, high and very high risk depending on the proportion of subjects having risk factor(s), the number of risk factors in a given population, and the type of risk factor (some risk factors may weigh twice the other ones). Next, we will ask reviewers to rate the applicability of a given study population based on their risk status in addition to other elements already considered in the QUADAS-2 tool as: 1) no concern, 2) serious concern, and 3) very serious concern. The answer to this additional question will feed into the overall rating of the patient selection “applicability Judgment” in answering the question, “Are there concerns that the included patients do not match the review question?”. Both “serious concern” and “very serious concern” will be interpreted as “high concern” in answering the stated question in QUADAS-2 tool.
2. **Reference Standard:** given that there is no agreed upon gold standard for diagnosis of PJI, we will consider the risk of bias of a reference standards as “low risk” in a given study if any of the followings were used:

- Joint fluid (with cell count, Gram stain, and culture) or tissue (with histopathology and culture). Note that Gram stain as a standalone test will not be considered a “low risk” reference standard.
- Presence of discharge of pus through the surgical wound or as a fistula at any time after surgery
- Presences of draining sinus tract or abscess; sinus tract if it communicates with the joint
- Presence of a systemic infection with pain in the joint and purulent fluid within the joint

1. **Flow and Timing:** If the population in an included study has received antibiotics, and/or chemotherapy before administration of index and/or reference standard tests, the risk of bias judgment for this domain will be considered as “high risk”. In case of revision surgery, there should be a washout period of at least 2 weeks between the administration of antibiotic and re-sampling.
